# Is Opium a Real Risk Factor for Esophageal Cancer or Just a Methodological Artifact? Hospital and Neighborhood Controls in Case-Control Studies

**DOI:** 10.1371/journal.pone.0032711

**Published:** 2012-03-01

**Authors:** Ramin Shakeri, Farin Kamangar, Dariush Nasrollahzadeh, Mehdi Nouraie, Hooman Khademi, Arash Etemadi, Farhad Islami, Hajiamin Marjani, Saman Fahimi, Alireza Sepehr, Atieh Rahmati, Christian C. Abnet, Sanford M. Dawsey, Paul Brennan, Paolo Boffetta, Reza Malekzadeh, Reza Majdzadeh

**Affiliations:** 1 Digestive Disease Research Center, Shariati Hospital, Tehran University of Medical Sciences, Tehran, Iran; 2 Division of Cancer Epidemiology and Genetics, National Cancer Institute, Bethesda, Maryland, United States of America; 3 Department of Public Health Analysis, School of Community Health and Policy, Morgan State University, Baltimore, Maryland, United States of America; 4 Department of Medical Epidemiology and Biostatistics, Karolinska Institute, Stockholm, Sweden; 5 Department of Internal Medicine and Center for Sickle Cell Disease, Howard University, Washington, District of Columbia, United States of America; 6 International Agency for Research on Cancer, Lyon, France; 7 University of Cambridge, Cambridge, United Kingdom; 8 Department of Pathology, BIDMC, Harvard Medical School, Boston, Massachusetts, United States of America; 9 Gastrointestinal and Liver Disease Research Center, Firoozgar Hospital, Tehran University of Medical Sciences, Tehran, Iran; 10 The Tisch Cancer Institute, Mount Sinai School of Medicine, New York, New York, United States of America; 11 International Prevention Research Institute, Lyon, France; 12 Department of Epidemiology and Biostatistics, School of Public Health, Tehran University of Medical Sciences, Tehran, Iran; 13 Knowledge Utilization Research Center (KURC), Tehran University of Medical Sciences, Tehran, Iran; Bremen Institute of Preventive Research and Social Medicine, Germany

## Abstract

**Background:**

Control selection is a major challenge in epidemiologic case-control studies. The aim of our study was to evaluate using hospital versus neighborhood control groups in studying risk factors of esophageal squamous cell carcinoma (ESCC).

**Methodology/Principal Findings:**

We compared the results of two different case-control studies of ESCC conducted in the same region by a single research group. Case definition and enrollment were the same in the two studies, but control selection differed. In the first study, we selected two age- and sex-matched controls from inpatient subjects in hospitals, while for the second we selected two age- and sex-matched controls from each subject's neighborhood of residence. We used the test of heterogeneity to compare the results of the two studies. We found no significant differences in exposure data for tobacco-related variables such as cigarette smoking, chewing Nass (a tobacco product) and hookah (water pipe) usage, but the frequency of opium usage was significantly different between hospital and neighborhood controls. Consequently, the inference drawn for the association between ESCC and tobacco use did not differ between the studies, but it did for opium use. In the study using neighborhood controls, opium use was associated with a significantly increased risk of ESCC (adjusted OR 1.77, 95% CI 1.17–2.68), while in the study using hospital controls, this was not the case (OR 1.09, 95% CI 0.63–1.87). Comparing the prevalence of opium consumption in the two control groups and a cohort enrolled from the same geographic area suggested that the neighborhood controls were more representative of the study base population for this exposure.

**Conclusions/Significance:**

Hospital and neighborhood controls did not lead us to the same conclusion for a major hypothesized risk factor for ESCC in this population. Our results show that control group selection is critical in drawing appropriate conclusions in observational studies.

## Introduction

Case-control studies are the design of choice in studying less common diseases such as esophageal cancer. Although esophageal cancer ranks 8^th^ in incidence amongst all cancers [1], it is rare enough that even in many large cohorts, it may take a long time to have enough numbers of cases sufficient for statistical analysis [2], [3], [4]. Therefore, although consortia of cohorts can help to have enough numbers of cases, case-control studies are still widely used to study the etiology of esophageal cancer.

Defining an appropriate sampling frame from which controls should be selected is arguably one of the most difficult tasks in designing a case-control study. The aim is to select a group of controls which are representative of the community from which cases have been selected. In their review of the methodological issues of case-control studies, Wacholder and colleagues have stressed the importance of study base and control selection in case-control studies, and discussed several sources for control selection, including population controls, hospital or disease registry controls, controls from a medical practice, friend controls, relative controls, controls selected from case series, proxy respondents and deceased controls [5]. Neighborhood and hospital-based controls have been used in many studies. Each of these controls has advantages and disadvantages. For example, enrolling hospital controls is usually more convenient and less costly, and information collected from cases and controls is more comparable in the sense that both cases and controls respond in a medical setting, but it also has the disadvantage that cases and controls may not be from the same study base and the referral pattern for the disease of interest may be different. A comprehensive treatment of this subject is given elsewhere [6].

The Golestan Case-Control Study in northeastern Iran was carried out in two phases. In the pilot phase of the study, 130 incident esophageal squamous cell carcinomas (ESCCs) and 260 matched hospital controls were enrolled, while in the main phase of the study, 300 ESCC cases and 571 matched neighborhood controls were recruited [7]. In this manuscript, we compare the results obtained from the pilot phase of this study, which used hospital controls, and the results of the main phase, which used neighborhood controls, to evaluate tobacco-related variables and opium as risk factors for ESCC.

## Methods

### Ethics Statement

The study was approved by the Institutional Review Boards of the Digestive Disease Research Center of Tehran University of Medical Sciences and the US National Cancer Institute.

### Case Selection

This study compares results from the pilot phase (March 2002–November 2003) and the main phase (December 2004–June 2007) of the Golestan Case-Control Study. Case selection and procedures in the pilot phase and the main phase of the study were the same. A detailed description of the case selection procedures has been published [8]. All cases were evaluated at Atrak Clinic in Khatam Hospital, located in Gonbad City, which is the only specialized clinic for upper gastrointestinal tract cancers in Golestan Province. Patients suspected of having upper gastrointestinal tract cancers were referred by local physicians to the clinic. A population-based cancer registry confirmed that about 70% of incident ESCC cases visited Atrak. All suspected cases underwent upper gastrointestinal endoscopy. Biopsy samples of the esophagus taken during endoscopy were reviewed by expert pathologists at the Digestive Disease Research Center, Tehran University of Medical Sciences. All cases enrolled in both phases had pathology-proven esophageal squamous cell carcinoma.

### Control Selection

#### Hospital-based controls in the pilot phase study

Hospitalized subjects were individually matched to each case on age and sex. Because tobacco use, alcohol consumption, and diet are thought to be important risk factors for ESCC [9], controls were selected from inpatient subjects with diseases thought to be unrelated to tobacco use, alcohol consumption, or diet. When a case was diagnosed, the control selection team reviewed the list of the patients in hospital wards, especially trauma wards, prepared a roster of potential sex- and age-matched (±2 years) controls and randomly selected two patients. If either of the selected controls refused or was too ill to participate, the team selected another control from the roster. Control selection matched the expected pattern of cases presenting to the local hospitals. Most (78.5%) of the controls were selected from Khatam hospital, where Atrak Clinic is located, and the rest of the controls were selected from other hospitals in Gonbad City. The response rate for hospital controls was 95% of the first selected patients.

#### Neighborhood controls in the main phase study

Neighborhood controls were selected using the Iranian Family Health Census as the sampling frame. Two subjects were matched to each case by place of residence (urban neighborhood or village), age (±2 years), and sex. The interview team identified all of the potentially eligible controls in the case's village or urban area, and randomly selected two subjects to interview. If either of the selected controls could not be interviewed for any reason, another person on the list was randomly invited, and so forth. The total number of enrolled neighborhood controls was 571. Of the enrolled neighborhood controls 77% were the first randomly selected subjects, 11% were the second, and the remainder required more than two selections. In nearly all instances the reason that an eligible control did not participate in the study was the absence of the control at the time of invitation [10].

### Data Collection

After obtaining written informed consent, a structured questionnaire with closed questions and pre-categorized responses was administered to both cases and controls by physician-researchers from Atrak Clinic. The questionnaire included detailed information on demographics, family history of cancer, history of tobacco, opium and alcohol use, drinking tea habits, oral health, and socioeconomic variables. More detailed information on the questionnaire is available elsewhere [8].

### Statistical Analysis

Univariate and multiple variable conditional logistic regression models were used to measure crude and adjusted odds ratios (OR) and 95% confidence Intervals (CI). In addition to the matching factors of age, sex, and place of residence, we also adjusted for education (as a determinant of socioeconomic status), ethnicity (Turkmen versus non-Turkmen) and consumption of cigarette, hookah, Nass and opium (for those analyses that these were not the main independent variable). We chose two previously identified risk factors for ESCC for comparing the two studies, use of different forms of tobacco and use of opium. We calculated the p-value of the heterogeneity test for each set of adjusted ORs. For significant differences between the two sets of controls, we also compared data from our two controls series with the data from the Golestan Cohort Study, a large cohort study conducted in the same geographic area [11], [12]. Age, sex and ethnicity distributions of the two control sets and the cohort were different from each other, so we used the cohort population as the standard population and used the indirect standardization method to calculate expected (standardized) prevalence rates for the other groups.

## Results

A total of 130 ESCC cases and 260 hospital-based controls were enrolled in the pilot phase of the study, while the corresponding numbers of cases and neighborhood controls in the main phase of the study were 300 and 571, respectively. Demographic characteristics of the studies participants are shown in **Table 1**.

**Table 1:** Demographic Characteristics of ESCC cases and controls in the two phases of the Golestan Case-Control Study of ESCC in Golestan Province, Iran.

	Hospital study		Neighborhood study		Difference in controls
	ESCCN (%)	ControlsN (%)	ESCCN (%)	ControlsN (%)	
**Total**	130	260	300	571	
**Gender**					
**Male**	79 (61)	158 (61)	150 (50)	278 (49)	P value<0.05
**Female**	51 (39)	102 (39)	150 (50)	293 (51)	
**Age**					
**< = 50**	22 (17)	40 (15)	32 (11)	61 (11)	P value<0.05
**51–60**	29 (22)	60 (23)	81 (27)	144 (25)	
**61–70**	38 (29)	83 (32)	86 (29)	177 (31)	
**>70**	41 (32)	77 (30)	101 (34)	189 (33)	
**Ethnicity**					
**Turkmen**	72 (55)	94 (36)	171 (57)	312 (55)	P value<0.05
**Non-Turkmen**	58 (45)	166 (64)	129 (43)	259 (45)	
**Residence**					
**Urban**	37 (29)	109 (42)	82 (27)	150 (26)	P value<0.05
**Rural**	93 (71)	151 (58)	218 (73)	421 (74)	

Distributions of age, ethnicity, and place of residence were similar between the cases in the two studies, but hospital-based controls were more likely to be non-Turkmen and live in the urban areas than the cases or the neighborhood controls.

**Tables 2** **,** **3** **,** **4** **,** **5** **,** **6** show the exposure distributions among cases and controls in the two phases of the study. Crude and adjusted ORs and 95% CIs are presented for each phase of the study. **Table 2** shows the results for smoking cigarettes. The striking feature of this table and the related results is the overall very low prevalence of smoking in this population, across cases, hospital controls and population controls and these rates were lower in the hospital-based study; 13% of the controls in the hospital-based study and 17% of the controls in the neighborhood-based study smoked cigarettes. However, the ORs were comparable in the two studies. Both studies showed an increase in the risk of ESCC with smoking, with an adjusted OR of less than 1.5. They also showed a dose-response trend with cumulative use of cigarettes.

**Table 2:** Cigarette use and risk of ESCC in cases and controls in the two phases of the Golestan Case-Control Study of ESCC in Golestan Province, Iran.

	Hospital study				Neighborhood study				P for heterogeneity adjusted ORs
	ESCC%	Control%	CrudeOR (95% CI)	Adjusted*OR (95% CI)	ESCC%	Control%	CrudeOR (95% CI)	Adjusted*OR (95% CI)	
**Cigarette Smoking**									
Never	107(82)	226(87)	1.00	1.00	232(78)	471(83)	1.00	1.00	
Ever	23(18)	34(13)	1.48 (0.80–2.75)	1.33(0.67–2.64)	67(22)	99(17)	1.35 (0.92–2.00)	1.05(0.67–1.64)	0.59
**Average amount (cigarettes per day)**									
Never Used	107(82)	226(87)	1.00	1.00	232(78)	471(83)	1.00	1.00	
< = Median**	10(8)	20(8)	1.09 (0.48–2.49)	1.04(0.42–2.61)	22(7)	49(9)	0.89 (0.51–1.56)	0.75(0.41–1.38)	0.56
>Median	13(10)	14(5)	2.06 (0.90–4.71)	1.71(0.68–4.27)	45(15)	50(9)	1.84 (1.13–2.99)	1.38(0.80–2.37)	0.70
**Duration**									
Never Used	107(82)	226(87)	1.00	1.00	232(78)	471(83)	1.00	1.00	
< = Median	16(12)	19(7)	1.96 (0.90–4.28)	1.64(0.72–3.69)	30(10)	49(9)	1.18 (0.72–1.94)	0.88(0.50–1.52)	0.29
>Median	7(6)	15(6)	0.99 (0.38–2.55)	0.94(0.33–2.62)	37(12)	50(9)	1.58 (0.95–2.62)	1.27(0.73–2.23)	0.58
**Cumulative** ***									
Never Used	107(82)	226(87)	1.00	1.00	232(78)	471(83)	1.00	1.00	
< = Median	10(8)	17(6.5)	1.30 (0.55–3.07)	1.22(0.47–3.19)	30(10)	49(9)	1.22 (0.74–2.01)	1.00(0.58–1.73)	0.73
>Median	13(10)	17(6.5)	1.66 (0.76–3.62)	1.42(0.59–3.40)	37(12)	50(9)	1.52 (0.91–2.53)	1.11(0.62–1.98)	0.66
**Age Started**									
Never Used	107(82)	226(87)	1.00	1.00	232(78)	471(83)	1.00	1.00	
>Median	10(8)	17(6.5)	1.26 (0.54–2.91)	1.01(0.41–2.48)	23(8)	49(9)	0.98 (0.57–1.68)	0.84(0.47–1.50)	.75
< = Median	13(10)	17(6.5)	1.76 (0.76–4.06)	1.75(0.72–4.25)	44(15)	50(9)	1.75 (1.08–2.84)	1.28(0.74–2.20)	.58

* Adjusted for opium, nass, hookah, ethnicity, education and place of residence.

** Median of control group in each set.

*** Cumulative = duration×amount.

**Table 3:** Nass use and risk of ESCC in cases and controls in the two phases of the Golestan Case-Control Study of ESCC in Golestan Province, Iran.

	Hospital study				Neighborhood study				P for heterogeneity adjusted ORs
	ESCC%	Control%	CrudeOR (95% CI)	Adjusted*OR (95% CI)	ESCC%	Control%	CrudeOR (95% CI)	Adjusted*OR (95% CI)	
**Nass**									
Never	110(85)	237(91)	1.00	1.00	256(85)	523(92)	1.00	1.00	
Ever	20(15)	23(9)	1.89 (0.98–3.62)	1.82(0.89–3.70)	44(15)	48(8)	2.09 (1.28–3.42)	1.53(0.88–2.64)	0.71
**Average amount (times per day)**									
Never	110(85)	237(91)	1.00	1.00	256(85)	523(92)	1.00	1.00	
< = Median**	11(8)	12(5)	2.05 (0.85–4.93)	1.93(0.76–4.92)	17(6)	24(4)	1.59 (0.78–3.25)	1.11(0.52–2.39)	0.42
>Median	9(7)	11(4)	1.74 (0.71–4.22)	1.71(0.66–4.40)	27(9)	24(4)	2.52 (1.38–4.61)	1.90(0.98–3.66)	0.85
**Duration**									
Never	110(85)	237(91)	1.00		256(85)	523(92)	1.00	1.00	
< = Median	10(7.5)	13(5)	1.65 (0.67–4.06)	1.43(0.54–3.76)	26(9)	24(4)	2.44 (1.32–4.49)	1.69(0.87–3.26)	0.77
>Median	10(7.5)	10(4)	2.19 (0.85–5.65)	2.35(0.86–6.41)	18(6)	24(4)	1.69 (0.83–3.41)	1.33(0.62–2.81)	0.43.
**Cumulative** ***									
Never	110(85)	237(91)	1.00	1.00	256(85)	523(92)	1.00	1.00	
< = Median	9(7)	12(5)	1.61 (0.64–4.04)	1.42(0.50–3.95)	22(7)	25(4)	2.19 (1.08–3.73)	1.36(0.70–2.66)	0.95
>Median	11(8)	11(4)	2.20 (0.89–5.39)	2.25(0.86–5.85)	22(7)	23(4)	2.20 (1.15–4.21)	1.74(0.86–3.53)	.68
**Age Started**									
Never	110(85)	237(91)	1.00	1.00	256(85)	523(92)	1.00	1.00	
>Median	12(9)	12(5)	1.56 (0.60–4.02)	1.65(0.59–4.55)	25(8)	27(5)	2.07 (1.06–4.04)	1.49(0.73–3.04)	0.88
< = Median	8(6)	11(4)	2.20 (0.93–5.18)	1.95(0.80–4.74)	19(6)	21(4)	2.11 (1.13–3.94)	1.56(0.79–3.06)	0.70

* Adjusted for cigarette smoking, opium and hookah consumption, ethnicity, education and place of residence.

** Median of control group in each set.

*** Cumulative = duration×amount.

**Table 4:** Hookah use in cases and controls of the two phases of the Golestan Case-Control Study of ESCC in Golestan Province, Iran.

	Hospital study				Neighborhood study				P for heterogeneity adjusted ORs
	ESCC%	Control%	CrudeOR(95% CI)	Adjusted**OR (95% CI)	ESCC%	Control %	CrudeOR (95% CI)	Adjusted**OR (95% CI)	
**Hookah**									
Never	119(92)	243(93)	1.00	1.00	280(93)	548(96)	1.00	1.00	
Ever	11(8)	17(7)	1.33 (0.59–3.00)	2.15(0.91–5.08)	20(7)	23(4)	1.81 (0.95–3.43)	1.79(0.90–3.55)	0.79
**Average amount (times per day)**									
Never Used	119(92)	243(93)	1.00	1.00	280(93)	548(96)	1.00		
< = Median**	5(4)	13(5)	0.81 (0.27–2.35)	1.33(0.44–3.98)	11(4)	18(3)	1.29 (0.59–2.84)	1.30(0.56–3.03)	0.97
>Median	6(4)	4(2)	2.94 (0.82–10.5)	5.78(1.25–26.59)	9(3)	5(1)	3.51 (1.17–10.5)	3.23(1.04–9.96)	0.60
**Duration**									
Never Used	119(92)	243(93)	1.00	1.00	280(93)	548(96)	1.00	1.00	
< = Median	9(7)	9(4)	1.91 (0.75–4.85)	3.43(1.25–9.43)	15(5)	12(2)	2.52 (1.14–5.55)	2.39(1.03–5.57)	0.61
>Median	2(1)	8(3)	0.55 (0.11–2.64)	0.77(0.15–3.87)	5(2)	11(2)	1.00 (0.34–2.90)	1.10(0.37–3.30)	0.70
**Cumulative** ***									
Never Used	119(92)	243(93)	1.00	1.00	280(93)	548(96)	1.00	1.00	
< = Median	9(7)	9(4)	1.93 (0.76–4.89)	3.26(1.20–8.80)	12(4)	12(2)	2.07 (0.87–4.88)	1.95(0.77–4.87)	0.48
>Median	2(1)	8(3)	0.50 (0.09–2.55)	0.77(0.14–4.06)	8(3)	11(2)	1.55 (0.62–3.87)	1.64(0.63–4.25)	0.39
**Age Started**									
Never Used	119(92)	243(93)	1.00	1.00	280(93)	548(96)	1.00	1.00	
>Median	4(3)	9(4)	0.92 (0.27–3.16)	1.29(0.36–4.54)	7(2)	11(2)	1.55 (0.62–3.87)	1.26(0.45–3.48)	0.97
< = Median	7(5)	8(3)	1.74 (0.62–4.81)	3.26(1.08–9.76)	13(4)	12(2)	2.22 (0.99–5.00)	2.33(0.97–5.60)	0.65

* Adjusted for cigarette smoking, opium, nass, ethnicity, education and place of residence.

** Median of control group in each study.

*** Cumulative = duration×amount.

**Table 5:** Opium use and risk of ESCC in cases and controls in the two phases of the Golestan Case-Control Study of ESCC in Golestan Province, Iran.

	Hospital study				Neighborhood study				P for heterogeneity adjusted ORs
	ESCC%	Control%	CrudeOR(95% CI)	Adjusted** OR(95% CI)	ESCC%	Control %	CrudeOR(95% CI)	Adjusted**OR(95% CI)	
**Opium**									
Never	85(65)	187(72)	1.00	1.00	210(70)	465(81)	1.00	1.00	
Ever	45(35)	73(28)	1.37 (0.85–2.21)	1.09(0.63–1.87)	90(30)	106(18)	1.95 (1.36–2.78)	1.77(1.17–2.68)	0.15
**Duration**									
Never	85(65)	187(72)	1.00	1.00	210(70)	465(81)	1.00	1.00	
< = Median**	27(21)	36(14)	1.69 (0.94–3.05)	1.48(0.78–2.81)	34(11)	53(9)	1.44 (0.88—2.39)	1.44(0.84–2.45)	0.94
>Median	18(14)	3714	1.05 (0.54–2.03)	0.73(0.35–1.51)	56(19)	53(9)	2.37 (1.54—3.65)	2.12(1.28–3.50)	0.02
**Age Started**									
Never	85(65)	187(72)	1.00	1.00	210(70)	465(81)	1.00	1.00	
>Median	26(20)	39(15)	1.33 (0.74–2.41)	1.07(0.54–2.10)	41(14)	53(9)	1.26 (0.74—2.16)	1.25(071–2.18)	0.72
< = Median	19(15)	34(13)	1.42 (0.73–2.7)	1.11(0.55–2.27)	49(16)	53(9)	2.5 (1.63—3.84)	2.32(1.40–3.82)	0.09

* Adjusted for cigarette smoking, nass, hookah, ethnicity, education and place of residence.

** Median of control group in each study.

**Table 6:** Determinants of opium use in hospital and neighborhood controls in the two phases of the Golestan Case-Control Study in Golestan Province, Iran.

	Hospital*		Neighborhood*		Combined**	
	OR	95% CI	OR	95% CI	OR	95% CI
**sex**						
male	1		1		1	
female	0.19¶	0.08–0.48	0.88	0.47–1.67	0.56†	0.34–0.92
**age**						
Above 70	1		1		1	
60–70	1.04	0.46–2.34	0.96	0.53–1.74	0.93	0.58–1.48
50–60	0.87	0.34–2.25	1.14	0.57–2.27	1	0.58–1.72
30–50	1.57	0.43–5.73	0.52	0.17–1.60	0.64	0.29–1.42
**ethnicity**						
Turkmen	1		1		1	
non-Turkmen	0.87	0.42–1.76	2.42¶	1.42–4.13	1.74‡	1.15–2.65
**place of residence**						
urban residence	1		1		1	
rural residence	2.18†	1.02–4.67	1.82	0.97–3.45	1.90‡	1.18–3.06
**marital status**						
Married	1		1		1	
widowed/divorced	0.75	0.29–1.93	1.49	0.74–3.01	1.20	0.69–2.09
**education**						
illiterate	1		1		1	
elementary	0.78	0.31–1.94	1.48	0.69–3.19	1.20	0.68–2.12
middle school and above	0.29	0.05–1.78	0.62	0.20–1.91	0.56	0.22–1.39
**home ownership**						
owned	1		1		1	
tenant	0.83	0.16–4.36	2.2	0.52–9.34	1.16	0.39–3.51
**Smoking**						
never smoked	1		1		1	
smoker	8.92¶	3.40–23.45	12.30¶	6.59–22.98	9.34¶	5.76–15.17
**History of chronic diseases**						
Absent	1		1		1	
Present	2.06	0.98–4.34	1.32	0.80–2.20	1.36	0.91–2.05
**Source of controls**						
Hospital	-	-	-	-	1	
Neighborhood	-	-	-	-	0.49¶	0.32–0.75

* Model includes all the variables in the table except source of controls;

** model includes all the variables in the table.

OR: odds ratio; CI: Confidence interval;

† p<0.05;

‡ p<0.01;

¶ p<0.001.

Table 3 shows the results for Nass use. The results were nearly identical in the two studies. Approximately 8 to 9% of the controls used Nass in each study, and in both, the adjusted ORs show that Nass use was associated with a 1.5–1.8-fold increased risk of ESCC.

The results for hookah are shown in **Table 4**. Only 7% of the controls in the pilot phase of study and 4% of the controls in the main phase of the study used hookah. The point estimates for the adjusted ORs were 1.8–2.1.

**Table 5** shows the results for opium use. Twenty-eight percent of the hospital-based controls but only 18% of the neighborhood-based controls reported using opium. However, the percentage of ESCC patients who used opium was quite similar in the two phases of the study (35% and 30%, respectively) (P value>0.05). The adjusted ORs were greater than 1 in both studies, while the OR was significant and much higher in the neighborhood-based study (OR 1.77) than in the hospital-based study (OR 1.09), it may make sense even though test of heterogeneity is not significant. Additionally, duration of use showed a dose-response association with ESCC risk in the neighborhood-based study, whereas it did not show such an association in the hospital-based study. In the hospital-based study, the questionnaire did not include average amount of opium used each day, so data on average amount and cumulative exposure were not available.

Calculated standardized opium consumption prevalence's were 0.17, 0.16 and 0.23 for the cohort subjects, the neighbourhood controls and the hospital controls, respectively. We also pooled the data of the 430 cases from the two phases of the study and compared this pooled case data with that of the neighborhood controls and the hospital based controls separately, using non-conditional logistic regression. The results showed that in the analysis of the 430 cases and 570 neighbourhood controls, after adjusting for the confounding factors, using opium had a significant association with ESCC (OR = 2.05, 95% CI 1.43–2.93) while in the analysis of the 430 cases and 260 hospital controls, it did not (OR = 0.87, 95% CI 0.57–1.33).

Table 6 shows that among both sets of controls, that smoking is by far the strongest determinant of opium use. Smoking is also a very strong determinant of ESCC, and so opium must be considered a confounder of the relationship between smoking and ESCC, and vice versa. Considering the results of table 6 we add a multivariable analyses in table 5, assessing opium as a risk factor for ESCC which has been adjusted for smoking. The results showed that the observed association with opium use cannot be explained by confounding effect of smoking.

## Discussion

In this study, we compared the associations of tobacco-related variables and opium use with ESCC risk in two phases of a case-control study in the same population, one phase using hospital controls and one using neighbourhood controls. We found that the results were similar for cigarette smoking and Nass consumption in the two phases of study; both showed a similar magnitude of increased ESCC risk. It was difficult to compare the results for hookah use, as the prevalence of consumption in the study area is very low and our sample size was modest. We found a notable difference between the pilot and main phase results, however, when examining the effect of opium use. Compared to the neighbourhood controls, hospital-based controls were more likely to use opium, at rates close to those seen in cases. Therefore, while the neighborhood-based study showed an increased risk of ESCC with opium use, the hospital-based study did not.

Whether hospital-based or population-based controls better satisfy the comparability criteria has been long debated in epidemiologic texts and articles [13]. As Miller and colleagues have discussed, the answer may depend on the question(s) asked, and each study must evaluate the circumstances individually [14]. There are a number of studies which have compared these two methods of control selection. In a case-control study of cervical cancer, where the exposures were variables related to pregnancy, marital status, intercourse, and smoking, West and colleagues showed that hospital controls were more ‘case like’ than population controls for all exposures, and this led to underestimating the effects [15]. In a case-control study of diet and colorectal cancer, however, there were no significant differences in conclusions using hospital or population controls, so that for most analyses the authors combined the two series [16]. In another study, Sadetzki and colleagues mentioned that the possibility of selection bias should be taken into consideration whenever hospital controls are used [17]. In one other study [18], Infante-Rivard compared population and hospital controls to study risk factors of leukemia in children. From comparisons with population survey data and socioeconomic data, this researcher concluded that the study groups came from the same base population but the distribution of exposures in hospital controls was closer to that of cases than those of population controls, which resulted in ORs closer to null when using hospital controls [18].

In the current study, the prevalence of smoking among the hospital controls was close to that of the neighborhood controls, and also close to that found in the pilot phase of the Golestan Cohort Study in the same population [11]. The ORs in the two case-control studies were similar, and from both we would conclude that cigarette smoking is a risk factor for ESCC, albeit more modestly than it is in other populations [19]. The hospital controls were mostly selected from patients admitted for elective surgery (73%) or trauma (21%), but there were internal medicine patients (6%) too. These conditions were selected under the assumption that they were not related to smoking. Most of the surgery patients were hospitalized for benign prostatic hyperplasia (BPH) or hernia. , There is a possible but controversial protective effect of low dose smoking on BPH [20], but the association, if it exists, is small and would be unlikely to affect our results. There is also a report that has shown an association between smoking and hernia [21] but it needs to be investigate more to find out the real association between smoking and hernia.

Use of Nass in the hospital and neighborhood controls was also similar, but it was higher than what was found in the pilot phase of the Golestan Cohort Study pilot phase of the cohort study (19). However, we should not expect that the prevalence in the case-control and cohort studies should be similar, since Nass use is a function of sex, age, and ethnicity (it is most commonly seen in Turkmen men), and the case-control and cohort studies were dissimilar in these demographic variables.

The pattern of association with hookah use was reasonably similar in the two phases of the study. However, as we mentioned above, the number of people who used hookah was very small, so it is difficult to draw any meaningful conclusions.

The main difference between two studies was related to the association of opium consumption and ESCC risk. The cases in both studies had similar rates of opium consumption, but the hospital controls reported a higher prevalence of opium use than the neighborhood controls. So OR for opium use in neighbourhood control set is significant while it is not statistically significant in hospital control set and although test of heterogeneity was not significant, considering that the power of analysis in interaction test is not high enough we think that P value of 0.15 can be an issue for discussion.

There are several potential explanations for this difference in prevalence among these two control groups. Hospital controls may not be representative of the population because in this area opium has traditionally been used to treat pain and numerous ailments, including those which brought some of these controls to the hospital. In addition, a recent study has shown that regular users of opium are more prone to accidents [22]. This report is consistent with our finding that 48% of the hospital control patients admitted for trauma used opium. If either of these hypotheses is true, then using hospital controls would result in Berksonian bias and, in this instance, the estimation of opium risk would be biased toward the null. On the other hand the slight differences in ORs between the two studies can not necessarily be due to control selection as there are many differences between the two case-control studies. Different case groups and twice as many cases in the neighborhood study than the hospital-based study (the hospital-based estimates are less robust) can inflating opium exposure in this control group.

We compared standardized rates of opium use among the two sets of controls in the current study and the participants in the Golestan Cohort Study, which is the most representative survey that we have of the community from which the cases were selected, and we found that the prevalence of opium use in the cohort was much closer to that in the neighbourhood controls than it was to the prevalence of opium use in the hospital controls. This suggests that the neighbourhood controls were more representative of the study base than the hospital controls, at least for opium exposure. On the other hand, one might argue that the reported rates of opium consumption among neighborhood controls and participants in the cohort are lower than the real rates because they are not questioned in a medical setting and when they are sick, so they may not answer the questions as truthfully, and this may result in information bias. However, a recent study by our group showed that the responses given to the questions in the pilot phase of the cohort study, also in a non-medical setting, were very close to the results found by testing urine for markers of codeine and morphine [23], so it seems that the questionnaire provides valid responses in this setting.

This study has several strengths and limitations. Notable strengths, especially for the comparisons in this paper, were the high participation rates of both hospital and neighbourhood controls and the fact that the same team members interviewed all the cases and controls in both studies. One of the limitations is the modest sample size of the hospital-based study. Another is the fact that we cannot exclude the possibility that some of the exposures under study (particularly opium use) might be associated with the reasons that the hospital controls were hospitalized.

In summary, the results of this study show that neighbourhood controls were superior to hospital controls in assessing the risk of ESCC associated with opium exposure in this population. But, as shown in this and other studies, both hospital and neighbourhood controls can bring biases into an evaluation. Optimal control group selection requires considerable thought, and the best control group may be different in different studies.
